# Identification of Compounds from the Water Soluble Extract of *Cinnamomum cassia* Barks and Their Inhibitory Effects against High-Glucose-Induced Mesangial Cells

**DOI:** 10.3390/molecules180910930

**Published:** 2013-09-05

**Authors:** Qi Luo, Shu-Mei Wang, Qing Lu, Jie Luo, Yong-Xian Cheng

**Affiliations:** 1 State Key Laboratory of Phytochemistry and Plant Resources in West China, Kunming Institute of Botany, Chinese Academy of Sciences, Kunming 650201, Yunnan, China; 2 College of Pharmacy, Chengdu University of Traditional Chinese Medicine, Chengdu 610075, Sichuan, China; 3 College of Chinese Medicine, Guangdong Pharmaceutical University, Guangzhou 510006, Guangdong, China; 4 College of Pharmaceutical Sciences, Dali University, Dali 671002, Yunnan, China

**Keywords:** Chinese cinnamon, *Cinnamomum cassia*, Lauraceae, phenolic compounds, diabetic nephropathy

## Abstract

The difficulty of diabetic nephropathy (DN) treatment makes prevention the best choice. *Cinnamomum cassia* barks, known as Chinese cinnamon or Chinese cassia, is one of the most popular natural spices and flavoring agents in many parts of the World. Since previous reports indicated that Chinese cinnamon extract could be used for the treatment of diabetes, we proposed that this spice may be beneficial for the prevention of DN. However, the responsible compounds need to be further identified. In this study, we isolated three new phenolic glycosides, cinnacassosides A–C (**1-3**), together with fifteen known compounds from the water soluble extract of Chinese cinnamon. The structures of the new compounds were identified by comprehensive spectroscopic evidence. Eleven compounds (**6-9**, **11**, **13-18**) were isolated from this spice for the first time, despite extensive research on this species in the past, which added new facets for the chemical profiling of this spice. These isolates were purposely evaluated for their inhibitory effects on IL-6 and extracellular matrix production in mesangial cells which are definitely implicated in DN. The results showed that compounds **4-8** could inhibit over secretion of IL-6, collagen IV and fibronectin against high-glucose-induced mesangial cells at 10 µM, suggesting that Chinese cinnamon could be used as a functional food against DN.

## 1. Introduction

Diabetic nephropathy (DN) is a major complication of diabetes and a leading cause of end-stage renal disease (ESRD) [1]. For ESRD, the ultimate treatment is renal transplantation. However, due to the insufficient supply of kidneys, the patients who can benefit from renal transplantation are much less than those who should throughout the World. Quite a number of ESRD patients die during the surgery waiting period. Since DN is major factor of ESRD, retarding the progression of DN may be an alternative way to solve or alleviate the gap between kidney supply and demand. Unfortunately, the lack of efficient drugs or advanced technologies for DN in clinical practice makes it quite hard for DN patients to receive ideal treatment. Since it is very difficult to treat DN patients, earlier prevention appears to be more important to slow the progression of DN. Increasing clinical evidences showed that chronic inflammation, overproduction of extracellular matrix and reactive oxygen species in renal cells were implicated in the progression of DN [2,3]. Therefore, research targeting on these pathogenic factors may contribute to the prevention of DN.

**Figure 1:**
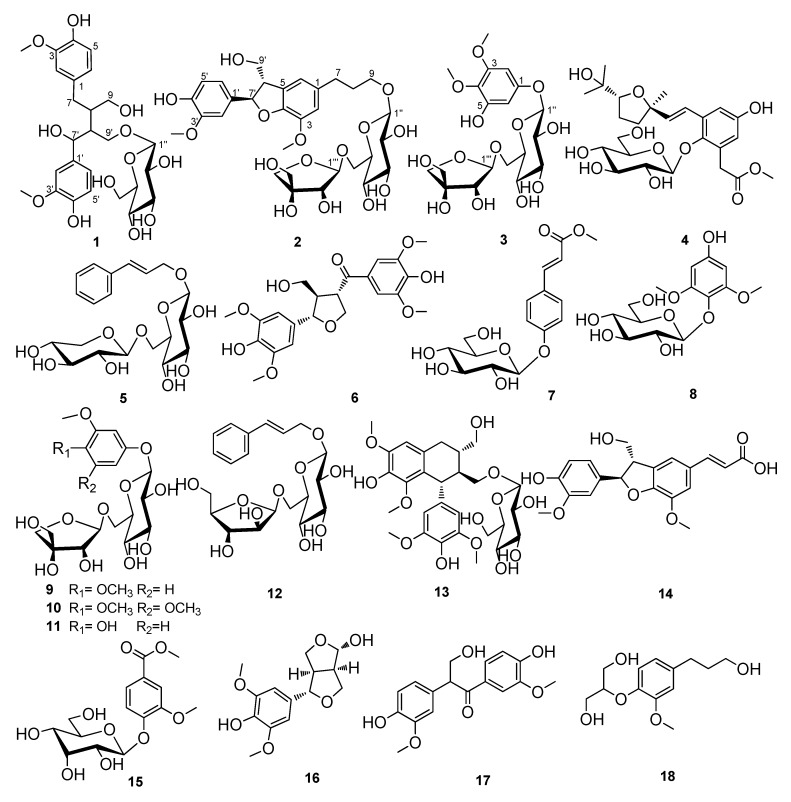
Structures of compounds **1**–**18***.*

For the prevention and control of chronic disease, functional foods will be the prior choice because they would be able to avoid adverse effects when taken regularly. Spices and a part of traditional herbs are considered to be important sources of functional foods. Spices have been used as flavoring agents across the World for thousands of years, indicating that spices are both common and safe food adjuncts [4]. Cinnamon, as a well-known spice comprising the types Ceylon cinnamon and cassia cinnamon (*C. cassia*) [5], is reported to be the second most important spice sold in the United States and European markets [6]. Due to the wide use of cinnamon, it has attracted much attention in the past [7]. Among these studies, many investigations on cinnamon have focused on type 2 diabetes. Polyphenols from cinnamon were reported to have *in vitro* and *in vivo* insulin-enhancing biological activity [8,9]. A human study showed that consuming cinnamon is beneficial for the health of patients with type 2 diabetes [10]. *C. cassia* (Lauraceae) is an evergreen tree originating in southern China (Guangdong, Guangxi and Yunnan Provinces), and now is widely cultivated there and southern and eastern Asia (Taiwan, Laos, Thailand, Vietnam, India, Indonesia and Malaysia) [11]. The dried stem bark of *C. cassia*, known as Chinese cinnamon or Chinese cassia is sold under the label cinnamon in the United States [12]. In China, it has been used as a spice, flavoring agent and preservative in food industry such as confectionery, desserts, pastries, and meat for a long history. Extensive phytochemical studies have been well conducted on *C. cassia* which revealed the presence of volatile oil, diterpenes [13,14], sesquiterpenoids [15], phenylpropanoids [16], flavonoids [17], and lignans [18]. The biological effects of *C. cassia* components, including antimicrobial [19], antioxidant [20], antiulcerogenic [21], anti-diabetic [22], and analgesic effects [23], are also well documented. We hypothesize that *C. cassia* could be very important for the prevention of chronic kidney diseases including DN according to its warm, spicy properties. Cinnamic aldehyde, the main constituent of *C. cassia* volatile oil, has been reported to be used to ameliorate metabolic disorder and relieve renal damage induced by diabetes via targeting Nrf2 activation [24], however, whether the other components, especially water soluble compounds in *C. cassia*, are beneficial for DN still remains unclear. As part of our continuous studies on the prevention and therapy of diabetic nephropathy, the water soluble fraction of the stem bark of *C. cassia* was investigated. Two new lignan glycosides (compounds **1** and **2**), a new phenolic glycoside **3** (Figure 1), were obtained, together with 15 known compounds **4**–**18**, and their inhibitory effects on DN were investigated using high-glucose-induced mesangial cells.

## 2. Results and Discussion

### 2.1. Structural Identification

**Figure 2:**
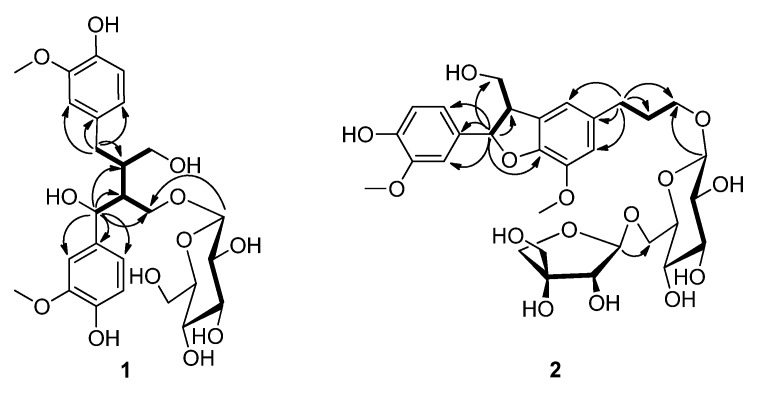
Key HMBC (→) and ^1^H-^1^H COSY (**—**) correlations of compounds **1** and **2**.

Compound **1** was obtained as a white solid. Its molecular formula was assigned as C_26_H_36_O_12_, with nine degrees of unsaturation, based on the HREIMS (*m/z* 540.2180 [M]^+^, calcd. for C_26_H_36_O_12_, 540.2207). The ^1^H-NMR spectrum of compound **1** (Table 1) revealed six aromatic protons [*δ*_H_ 6.74 (1H, d, *J* = 1.5 Hz, H-2), 6.66 (1H, d, *J* = 8.0 Hz, H-5), and 6.57 (1H, dd, *J* = 8.0, 1.5 Hz, H-6); 6.84 (1H, s, H-2′), and 6.70 (2H, d, *J* = 8.0 Hz, H-5′, H-6′)] and two methoxyl [*δ*_H_ 3.74, 3.73 (6H, s, 3-OCH_3_, 3′-OCH_3_)] signals. The ^13^C-NMR spectrum (Table 1) of **1** displayed 26 carbon signals, including one glucopyranosyl moiety [*δ*_C_ 103.2 (C-1′′), 73.6 (C-2′′), 76.9 (C-3′′), 70.1 (C-4′′), 77.0 (C-5′′), and 61.2 (C-6′′)], two benzene ring, two methoxyl, three methylene (two oxygenated), and three methine (one oxygenated) groups. The ^1^H- and ^13^C-NMR data of **1** were thus characteristic of a lignan glycoside, and was highly similar to those of 7-hydroxy-9′-*β*-glucopyranosyloxyl secoisolariciresinol [25]. The two compounds have the same molecular formula, indicating they were isomers, the only difference was that the hydroxyl group at C-7 in 7-hydroxy-9′-*β*-glucopyranosyloxyl secoisolariciresinol was moved to C-7′ in compound **1**, which was established by the HMBC correlations (Figure 2) from H-7′ (*δ*_H_ 4.72) to C-1′ (*δ*_C_ 134.5), C-2′ (*δ*_C_ 110.0), C-6′ (*δ*_C_ 118.3), C-8′ (*δ*_C_ 50.0), C-9′ (*δ*_C_ 66.8), and C-8 (*δ*_C_ 42.1) and correlations from H-7 (*δ*_H_ 2.83, 2.41) to C-1 (*δ*_C_ 131.9), C-2 (*δ*_C_ 112.7), C-6 (*δ*_C_ 120.6), C-8 (*δ*_C_ 42.1), and C-9′. The ^1^H-^1^H COSY correlations (Figure 2) of H-7′/H-8′, H-8′/H-9′, H-8′/H-8, H-8/H-7, and H-8/H-9 further confirmed this assumption. The HMBC correlation from H-1′′ (*δ*_H_ 4.17) to C-9′ confirmed the glucopyranosyl moiety was connected to C-9′. The presence of D-glucose was confirmed by TLC and optical rotation analyses. The configuration of glycosidic linkage of the glucopyranoside moiety in **1** was determined to be *β* on the basis of the *J* value of the anomeric proton signal at *δ*_H_ 4.17 (1H, d, *J* = 7.8 Hz, H-1′′). Although the ROESY correlations of H-8′/H-7a and H-7′/H_2_-7 were observed, however, it is not helpful for determining the relative configurations at C-8 and C-8′ because of the free rotation of the chain. Thus, the structure of **1** was established to be 7′-hydroxy-9′-*β*-glucopyranosyloxyl secoisolariciresinol, and the new compound was named cinnacassoside A.

Compound **2** gave a molecular formula of C_31_H_42_O_15_ from the HREIMS at *m/z* 654.2521 ([M]^+^, calcd for C_31_H_42_O_15_, 654.2524), corresponding to 11 degrees of unsaturation. The ^1^H-NMR spectrum of **2** (Table 1) showed an ABX coupling system [*δ*_H_ 6.95 (1H, d, *J* = 2.0 Hz, H-2′), 6.82 (1H, dd, *J* = 8.0, 2.0 Hz, H-6′), and 6.77 (1H, d, *J* = 8.0 Hz, H-5′)], two singlet aromatic protons [*δ*_H_ 6.76 (1H, s, H-2) and 6.75 (1H, s, H-6)], and two methoxyl [*δ*_H_ 3.86 (3H, s, 3-OCH_3_), 3.82 (3H, s, 3′-OCH_3_)] signals. The ^1^H- and ^13^C-NMR data (Table 1) of **2** showed 31 carbon signals, and were similar to those of symplolignanoside A [26], indicating a lignan glucoside with the same aglycon and saccharide moieties as the latter. Inspection of the ^13^C-NMR spectrum of compound **2** confirmed the presence of one glucopyranosyl [*δ*_C_ 104.7 (C-1′′), 75.3 (C-2′′), 77.0 (C-3′′), 71.8 (C-4′′), 78.2 (C-5′′), and 68.8 (C-6′′)] and one apiofuranosyl [*δ*_C_ 111.1 (C-1′′′), 78.1 (C-2′′′), 80.7 (C-3′′′), 75.1 (C-4′′′), and 65.6 (C-5′′′)] group in the molecule. The terminal apiofuranosyl unit was deduced to be attached at C-6′′ of the glucopyranosyl unit via oxygen on the basis of the downfield shift of C-6′′ (*δ*_C_ *Δ* = + 6.0 ppm) of the glucopyranosyl unit and the HMBC (Figure 2) correlation from H-6′′ (*δ*_H_ 3.98, 3.61) to C-1′′′. The glucopyranosyl unit was established to connect to C-9 of the aglycon by the HMBC correlation from H-1′′ (*δ*_H_ 4.24) to C-9 (*δ*_H_ 70.3). The two saccharide moieties were assigned to be *β*-form by the ^1^H-NMR spectroscopic data and the coupling constants of the anomeric proton signals at *δ*_H_ 4.24 (1H, d, *J* = 7.8 Hz, H-1′′) and 5.01 (1H, d, *J* = 2.4 Hz, H-1′′′). The HMBC correlations from H-7′ (*δ*_H_ 5.50) to C-1′ (*δ*_C_ 134.9), C-2′ (*δ*_C_ 110.6), C-6′ (*δ*_C_ 119.8), C-8′ (*δ*_C_ 55.6), C-9′ (*δ*_C_ 65.1), and C-4 (*δ*_C_ 147.6), and correlations from H-7 (*δ*_H_ 2.69) to C-1 (*δ*_C_ 137.1), C-2 (*δ*_C_ 114.2), C-6 (*δ*_C_ 118.1), C-8 (*δ*_C_ 33.2), and C-9′, along with the ^1^H-^1^H COSY correlations of H-7/H-8, H-8/H-9, H-7′/H-8′, and H-8′/H-9′ further confirmed the linkages of the two C_3_ units in compound **2** were the same as those in symplolignanoside A. The two methoxyl groups was deduced to be located at C-3 (*δ*_C_ 145.3) and C-3′ (149.2) by the HMBC correlations from 3-OCH_3_ to C-3 and from 3′-OCH_3_ to C-3′, respectively. The ^3^*J* value of H-7′ was 6.3 Hz, along with the ROESY correlation of H-7′/H-9′ indicated the *trans*-configuration of H-7 and H-8 [27,28]. The sugar moieties were assigned as d-glucose and d-apiose by TLC and optical rotation analyses. Thus, the structure of compound **2** was assigned as dihydrodehydrodiconiferyl alcohol-9-*β*-d-apiofuranosyl (1→6)-*β*-d-glucopyranoside, and it was named cinnacassoside B.

The molecular formula of compound **3** was deduced to be C_19_H_28_O_13_ by HREIMS at *m/z* 464.1519 ([M]^+^, calcd for C_19_H_28_O_13_, 464.1530). The ^1^H-NMR spectrum of **3** (Table 1) exhibited two overlapping single aromatic protons [*δ*_H_ 6.30 (2H, s, H-2, H-6)], two methoxyl [*δ*_H_ 3.80 (3H, s, 3-OCH_3_), 3.72 (3H, s, 4-OCH_3_)] and two anomeric protons [*δ*_H_ 4.75 (1H, d, *J* = 7.5 Hz, H-1′′), 4.98 (1H, d, *J* = 2.5 Hz, H-1′′′)] signals. The ^13^C-NMR spectrum (Table 1) of **3 ** displayed 19 carbon signals, including one benzene ring [*δ*_C_ 155.8 (C-1), 95.2 (C-2), 154.8 (C-3), 133.2 (C-4), 152.0 (C-5), and 99.1 (C-6)], one glucopyranosyl moiety [*δ*_C_ 103.0 (C-1′′), 74.9 (C-2′′), 78.0 (C-3′′), 71.7 (C-4′′), 77.1 (C-5′′) and 68.7 (C-6′′) and one apiofuranosyl group [*δ*_C_ 111.0 (C-1′′′), 78.0 (C-2′′′), 80.5 (C-3′′′), 75.0 (C-4′′′) and 65.7 (C-5′′′)], and two methoxyl [*δ*_C_ 56.5 (3-OCH_3_), 61.1 (4-OCH_3_)] signals. The ^1^H and ^13^C-NMR data of **3** were highly similar to those of 3,4-dimethoxyphenol *β*-d-apiofuranosyl (1→6)-*β*-d-glucopyranoside [26] except that a sp^2^ methine in 3,4-dimethoxyphenol *β*-d-apiofuranosyl (1→6)-*β*-d-glucopyranoside was replaced by a sp^2^ quaternary carbon in **3** and the downfield shift of C-5 (*δ*_C_ *Δ* = +37.8 ppm) and upfield shift of C-2 (*δ*_C_ *Δ* = −9.9 ppm), C-4 (*δ*_C_ *Δ* = −12.5 ppm) and C-6 (*δ*_C_ *Δ* = −12.4 ppm) in **3**, indicating there was an additional hydroxyl group connected to C-5 in **3**, in accordance with the molecular displayed by the HREIMS. The presence of glucose and apiose was confirmed by TLC and optical rotation comparison with authentic samples after acid hydrolysis and purification. Besides, the HMBC correlations from H-6 to C-1, C-4, and C-5 further confirmed this assumption. The HMBC correlations from *δ*_H_ 3.80 (3H, s, 3-OCH_3_) to C-3 and from *δ*_H_ 3.72 (3H, s, 4-OCH_3_) to C-4 indicated the two methoxyl groups were located at C-3 and C-4, respectively. The downfield shift of C-6′′ (*δ*_C_ 68.7), along with the HMBC correlations from H-1′′′ to C-6′′ indicated the apiofuranosyl moiety was connected to C-6′′ of the glucopyranosyl unit.

**Table 1:** The ^1^H and ^13^C-NMR spectroscopic data of **1-3**.

Position	1		2		3	
	*δ*_H_ (*J* in Hz)	*δ*_C_, mult	*δ*_H_ (*J* in Hz)	*δ*_C_, mult	*δ*_H_ (*J* in Hz)	*δ*_C_, mult
1		131.9, qC		137.1, qC		155.8, qC
2	6.74, d, 1.5	112.7, CH_2_	6.76, s	114.2, CH	6.30, s	95.2, CH
3		147.5, qC		145.3, qC		154.8, qC
4		144.6, qC		147.6, qC		133.2, qC
5	6.66, d, 8.0	115.4, CH		129.9, qC		152.0, qC
6	6.57, dd, 8.0, 1.5	120.6, CH	6.75, s	118.1, qC	6.30, s	99.1, CH
7	2.83, dd, 13.5, 4.4	32.2, CH_2_	2.69, dd, 13.8, 6.6	33.1, CH_2_		
	2.41, m					
8	2.57, m	42.1, CH	1.91, m	33.2, CH_2_		
9	3.85, t, 7.4	72.0, CH_2_	3.90, m	70.3, CH_2_		
	3.57, t, 7.4		3.54, m			
1′		134.5, qC		134.9, qC		
2′	6.84, d, 1.5	110.0, CH_2_	6.95, d, 2.0	110.6, CH		
3′		147.5, qC		149.2, qC		
4′		145.6, qC		147.6, qC		
5′	6.70, d, 8.0	115.1, CH	6.77, d, 8.0	116.2, CH		
6′	6.70, d, 8.0	118.3, CH	6.82, dd, 8.0, 2.0	119.8, CH		
7′	4.72, d, 6.0	81.9, CH	5.50, d, 6.3	89.2, CH		
8′	2.30, m	50.0, CH	3.48, m	55.6, CH		
9′	4.05, dd, 9.0, 7.8	66.8, CH_2_	3.86, m,	65.1, CH_2_		
	3.43, m		3.76, m			
1′′	4.17, d, 7.8	103.2, CH	4.24, d, 7.8	104.7, CH	4.75, d, 7.5	103.0, CH
2′′	2.79, m	73.6, CH	3.19, m	75.3, CH	3.75, d, 9.8	74.9, CH
3′′	3.10, m	76.9, CH	3.40, m	77.0, CH	3.92, m	78.0, CH
4′′	3.05, m	70.1, CH	3.28, m	71.8, CH	3.32, m	71.7, CH
5′′	3.12, m	77.0, CH	3.90, m	78.2, CH	3.56, m	77.1, CH
6′′	3.66, d,10.3	61.2, CH_2_	3.98, dd, 11.2, 1.8	68.8, CH_2_	4.03, d, 10.7	68.7, CH_2_
	3.43, m		3.61, m		3.60, m	
1′′′			5.01, d, 2.4	111.1, CH_2_	4.98, d, 2.5	111.0, CH
2′′′			3.33, m	78.1, CH_2_	3.42, d, 8.6	78.0, CH
3′′′				80.7, qC		80.5, CH
4′′′			3.96, d, 9.7	75.1, CH_2_	3.98, d, 9.6	75.0, CH_2_
			3.75, m		3.76, d, 7.4	
5′′′			3.56, m	65.6, CH_2_	3.58, 2H, m	65.7, CH_2_
3-OCH_3_	3.74, 3H, s	55.6, CH_3_	3.82, s	56.5, CH_3_	3.80, s	56.5, CH_3_
3′-OCH_3_	3.73, 3H, s	55.6, CH_3_	3.86, s	56.9, CH_3_		
4-OCH_3_					3.72, s	61.1, CH_3_

In the HMBC spectrum, the correlations from H-1′′ to C-1 indicated the glucopyranosyl unit was linked to C-1. The *β*-configurations of the two saccharide moieties were assigned by the coupling constants of the anomeric proton signals at *δ*_H_ 4.75 (1H, d, *J* = 7.5 Hz, H-1′′) and 4.98 (1H, d, *J* = 2.5 Hz, H-1′′′). Thus, the structure of **3** was determined as 3,4-dimethoxy- 5-hydroxyphenol *β*-d-apiofuranosyl (1→6)-*β*-d-glucopyranoside, named as cinnacassoside C.

The known compounds were identified as cinnacasside C (**4**) [29], rosavin (**5**) [30], (−)-(7′*S*,8*S*,8′*R*)-4,4′-dihydroxy-3,3′,5,5′-tetramethoxy-7′,9-epoxylignan-9′-ol-7-one (**6**) [31], lino- cinnamarin (**7**) [32], leonuriside (**8**) [33], 3,4-dimethoxyphenol *β*-d–apiofuranosyl (1→6)- *β*-d-glucopyranoside (**9**) [26], 3,4,5-trimethoxyphenol *β*-d–apiofuranosyl (1→6)-*β*-d-glucopyranoside (**10**) [21], 3-trimethoxy-4- hydroxyphenol *β*-d–apiofuranosyl (1→6)-*β*-d-glucopyranoside (**11**) [34],rosarin (**12**) [30], (−)-lyoniresinol 3*α-O-β*-d-glucopyranoside (**13**) [35], spicatolignan B (**14**) [36], methyl 3-methoxy-4-(*β*-d-allopyranosyloxy)benzoate (**15**) [37], 6-hydroxy-2-(4-hydroxy- 3,5-dimethoxyphenyl)-3,7-dioxabicyclo-[3.3.0]-octane (**16**) [38], evofolin B (**17**) [39], and 2-[4-(3-hydroxypropyl)-2-methoxyphenoxy]-1,3-propanediol (**18**) [40] by comparison of their spectroscopic data with those in the corresponding literature.

### 2.2. Inhibition of Fibronectin, Collagen IV, and IL-6 Secretion

**Figure 3:**
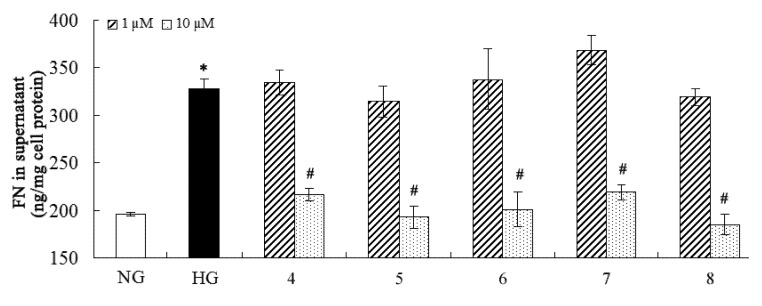
Inhibitory effect of the compounds on fibronectin. * *p* < 0.05 *vs*. normal glucose; ^#^ *p* < 0.05 *vs.* high glucose.

**Figure 4:**
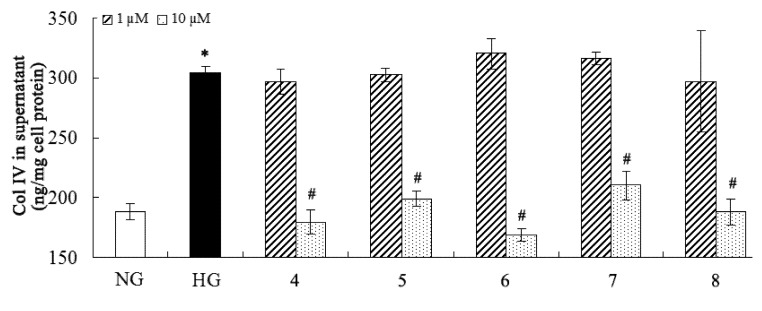
Inhibitory effect of the compounds on collagen IV. * *p* < 0.05 *vs*. normal glucose; ^#^ *p* < 0.05 *vs.* high glucose.

**Figure 5:**
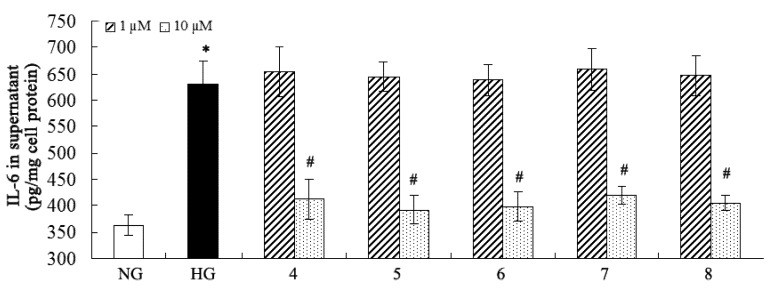
Inhibitory effect of the compounds on IL-6. * *p* < 0.05 *vs*. normal glucose; ^#^ *p* < 0.05 *vs.* high glucose.

Many pathogenic factors are involved in DN, and overproduction of extracellular matrix (such as fibronectin and collagen IV) and proinflammatory factors (such as IL-6) are distinctly implicated in DN [2,41]. Considering the traditional medical applications of *C. cassia*, the isolated compounds were purposely evaluated for their inhibitory effects on fibronectin, collagen IV, and IL-6 overproduction against high-glucose-induced mesangial cells (Figure 3, Figure 4 and Figure 5). The results showed that compounds **4-8** could inhibit overproduction of fibronectin, collagen IV, and IL-6 against high-glucose-induced mesangial cells at 10 µM, while all the other compounds were not active in this assay at the same concentration. Compound **4** is a novel natural hybrid with a unique geranylphenylacetate carbon skeleton which was previously isolated from the same material [29], however, its biological activities remained unknown yet.

This study revealed its possible role in DN. We noted that compounds **3**, **8**–**11** are structurally analogues, however, only **8** was found to be active in this assay. Although compounds **5** and **12** differed only from the presence of apiose or glucose, their bioactivity is different, **5** is active and **12** is inactive. Actually, compound **5** exhibited a broad range of biological effects such as on antidepressant, nootropic, anticancer, neurotropic, immunostimulating and hepatoprotective properties which have attracted great research interest in the past [42,43,44,45,46]. Oxidative stress in also one of the major pathogenic factors of DN, in general, the other phenolic compounds should possess antioxidant activity which will be beneficial for DN, however, this need to be further investigated.

## 3. Experimental

### 3.1. Equipment

Optical rotations were taken on a Horiba SEAP-300 polarimeter. UV spectra were obtained on a Shimadzu UV-2401PC (Shimadzu, Kyoto, Japan) spectrophotometer. 1D and 2D NMR spectra were recorded on a Bruker AM-400 or DRX-500 or Avance III 600 NMR spectrometer. Unless otherwise specified, chemical shifts (*δ*) were expressed in ppm with reference to the solvent signals. Mass spectra were obtained on a VG Auto Spec-3000 mass spectrometer (VG, Manchester, UK). Column chromatography (CC) was performed on silica gel (200-300 mesh, Qingdao Marine Chemical Inc., People’s Republic of China), RP-18 (40-60 μm, Daiso Co., Japan), MCI gel CHP 20P (75-150 μm, Tokyo, Japan), and Sephadex LH-20 (Amersham Biosciences, Sweden). Semipreparative HPLC was carried out using an Agilent 1200 liquid chromatograph, the column used was a 250 mm × 4.6 mm i.d., 5 μm, Zorbax SB-C_18_.

### 3.2. Plant Material

The *C. cassia* barks were collected at Yunnan Xianghui Biological Technology Co. Ltd. (YNXHBT) in July, 2010. The sample was identified by Mr. Fu-Shou Xie at YNXHBT. A voucher specimen (CHYX0173) was preserved at the State Key Laboratory of Phytochemistry and Plant Resources in West China, Kunming Institute of Botany, Chinese Academy of Sciences, China.

### 3.3. Extraction and Isolation

Dried powders of *C. cassia* barks (50 kg) were extracted three times with 80% EtOH (3 × 90 L) under reflux to give a residue (7.5 kg) after removal of solvent under reduced pressure. The residue was dissolve in acetone and then filtered, the acetone solution was evaporated in vacuum to get the acetone-soluble extract (5.0 kg), which was suspended in water and followed by successive partition with petroleum ether and EtOAc. The water-soluble extract (1.5 kg) was subjected to silica gel column chromatography (CC) (20 × 120 cm, 7.5 kg) and eluted with a isocratic of CHCl_3_-MeOH-H_2_O (80:20:2.5, 25 L, *v/v*) to yield three fractions, A–C. Fraction A (3.1 g) was submitted to RP-18 CC (3.0 × 3.0 cm, 80 g) eluted with gradient aqueous MeOH (10:90, 30:70, 50:50, 70:30, 90:10, 1L, each, *v/v*) to yield two fractions, A1 and A2. Fraction A1 (400 mg) was chromatographed over MCI gel CHP 20P CC (2.0 × 50 cm, 100 g) eluted with MeOH/H_2_O (20:80, 30:70, 40:60, 50:50, 60:40, 70:30, 500 mL, each, v/v) to afford two portions, A1a and A1b. Fraction A1a (85 mg) was purified by preparative TLC eluting with CHCl_3_/MeOH (10:1, 100 mL, *v/v*) to afford compound **17** (2.6 mg). Fraction A2 (1 g) was subjected to MCI gel CHP 20P CC (2.0 × 50 cm, 100 g) eluted with MeOH/H_2_O, (30:70, 40:60, 50:50, 60:40, 70:30, 80:20, 700 mL, each, *v/v*) to yield three fractions, A2a-A2c. Fraction A2b (20 mg) was purified by preparative TLC eluting with CHCl_3_/MeOH (11:1, 100 mL, *v/v*) to give compound **16** (3.5 mg). Fraction A2c (300 mg) was purified by preparative TLC eluting with CHCl_3_/MeOH (11:1, 100 mL, *v/v*) to give compound **6** (19.7 mg).

Fraction B (155 g) was subjected to Sephadex LH-20 CC (6.0 × 125 cm, 500 g) (MeOH) to obtain two portions, B1 and B2. Fraction B1 (5.2 g) was separated by RP-18 CC (3.0 × 50 cm, 200 g) eluted with MeOH/H_2_O (10:90, 30:70, 50:50, 70:30, 90:10, 1 L, each, *v/v*) to provide three portions, B1a-B1c. Fraction B1b (462 mg) was purified by preparative TLC eluting with CHCl_3_/MeOH/H_2_O (3:1:0.05, 200 mL, *v/v/v*) to obtain compounds **12** (3.8 mg) and **5** (3.9 mg), the residue of fraction B1b was further purified using successive semi-preparative HPLC (MeOH/H_2_O, 33:77, 5 L, *v/v*) to yield compounds **1** (9.2 mg), **2** (10.5 mg), and **13** (3.9 mg). Fraction B1c (240 mg) was purified by preparative TLC eluting with CHCl_3_/MeOH/H_2_O (6:1:0.05, 150 mL, *v/v/v*) to yield compounds **9** (30.8 mg) and **10 ** (42.8 mg). Fraction B2 (5.5 g) was subjected to CC over MCI gel CHP 20P (3.0 × 3.0 cm, 200 g) eluted with gradient aqueous MeOH (20:80, 30:70, 50:50, 70:30, 80:20, 1 L, each, *v/v*) to obtain three fractions, B2a–B2c. Fraction B2a (105 mg) was purified by preparative TLC eluting with CHCl_3_/MeOH/H_2_O (3:1:0.05, 100 mL, *v/v/v*) to yield compounds **11** (4.8 mg) and **15** (2.4 mg). Fraction B2b (201 mg) was purified using successive semi-preparative HPLC (MeOH/H2O, 20:80, 2 L) to yield compound **14** (1.0 mg).

Fraction C (1 g) was subjected to CC over MCI gel CHP 20P (3.0 × 3.0 cm, 150 g) eluted with gradient aqueous MeOH (MeOH/H_2_O, 10:90, 30:70, 50:50, 70:30, 80:20, 1 L, each, *v/v*) to yield three fractions, C1-C3. Fraction C1 (70 mg) was purified by preparative TLC eluting with CHCl_3_/MeOH (5:1, 100 mL, *v/v*) and then followed by successive semi-preparative HPLC (MeOH/H_2_O, 20:80, 5L) to yield compounds **3** (9.3 mg), **8** (4.3 mg) and **4** (1.1 mg). Fraction C2 (100 mg) was purified by preparative TLC eluting with CHCl_3_/MeOH (8:1, 150 mL, *v/v*) to provide compound **7** (3 mg). Fraction C3 (20 mg) was purified by preparative TLC eluting with CHCl_3_/MeOH (9:1, 100 mL, *v/v*) to obtain compound **18** (1.5 mg).

*Cinnacassoside A* (**1**). White solid; 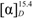 −31.9 (*c* 0.2, MeOH); UV (MeOH) *λ*_max_ (log *ε*): 203 (4.65), 226 (4.06), 281 (3.64) nm;^1^H (DMSO-*d*_6_, 600 MHz) and ^13^C-NMR (DMSO-*d*_6_, 150 MHz) data, see Table 1; EI-MS: *m/z* 540 [M]^+^; HREIMS: *m/z* 540.2180 [M]^+^ (calcd for C_26_H_36_O_12_, 540.2207).

*Cinnacassoside B* (**2**). Yellow solid; 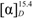 −38.0 (*c* 0.2, MeOH); UV (MeOH) *λ*_max_ (log *ε*): 205 (4.74), 283 (3.72) nm;^1^H (CD_3_OD, 600 MHz) and ^13^C-NMR (CD_3_OD, 150 MHz) data, see Table 1; EIMS: *m/z* 654 [M]^+^; HREIMS: *m/z* 654.2521 [M]^+^, calcd for C_31_H_42_O_15_, 654.2524).

*Cinnacassoside C* (**3**). Yellow solid; 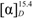 −82.6 (*c* 0.2, MeOH); UV (MeOH) *λ*_max_ (log *ε*): 204 (4.54), 271 (3.01) nm;^1^H (CD_3_OD, 600 MHz) and ^13^C-NMR (CD_3_OD, 125 MHz) data, see Table 1; EI-MS: *m/z* 464 [M]^+^; HREIMS: *m/z* 464.1519 [M]^+^ (calcd for C_19_H_28_O_13_, 464.1530).

### 3.4. Acid Hydrolysis of **1-3**

A solution of **1** (6 mg), **2** (8 mg), or **3** (8 mg) in 2 N HCl was heated at 90 °C on a water bath for 2 h. After cooling, the reaction mixture was extracted by EtOAc. The aqueous layer was neutralized with 2 N NaOH and dried by freeze-drying. The residue was purified by semipreparative HPLC with gradient aqueous MeOH to afford pure sugars. They were identified as d-glucose and d-apiose by comparison with authentic samples on TLC and measuring their optical rotations [glucose: EtOAC–MeOH–H_2_O (4:1:0.1), *R_f_* 0.2, 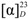 +37 (*c* 0.06, H_2_O); apiose: EtOAC–MeOH–H_2_O (4:1:0.1), *R_f_* 0.5, 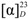 +5(*c* 0.05, H_2_O)].

### 3.5. Inhibition of Fibronectin, Collagen IV, and IL-6 Secretion

Rat mesangial cells were grown in Dulbecco’s modified Eagle’s medium (Invitrogen, Carlsbad, CA, USA) containing 5.6 mM D-glucose (pH 7.4; Sigma Chemical Co., St. Louis, MO, USA), supplemented with 20% fetal calf serum (FCS; Invitrogen), 100 U/mL penicillin, 100 μg/mL streptomycin, and 10 mM HEPES. After the mesangial cells reached 80% confluence, their growth was arrested in 0.5% FCS for 24 h. Exposure of the mesangial cells to medium containing high-concentration glucose induced the overproduction of fibronectin (FN), collagen IV (Col IV), and IL-6, as described in the previous reports [47,48]. To determine whether the compounds inhibited the FN, collagen IV, and IL-6 overproduction triggered by high glucose, the mesangial cells were pretreated with 1 or 10 μM of each compound for 1 h and then stimulated with high glucose (25 mM) for 24 h. The levels of supernatant FN, collagen IV, and IL-6 were measured with a solid-phase quantitative sandwich enzyme-linked immunosorbent assay (ELISA) kit for FN, collagen IV, and IL-6 (Uscn Life Science Inc., Wuhan, China). The concentration in the culture supernatant was normalized to the total amount of cell protein, quantified with the BCA method [41].

### 3.6. Statistics

The differences were tested using ANOVA. All values are expressed as mean ± S.D., and statistical significance was defined as *p* < 0.05.

## 4. Conclusions

**Figure 6:**
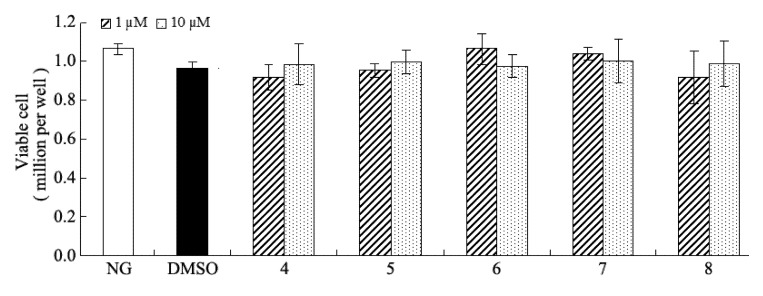
Effect of the compounds on cell viability at 1 or 10 µM.

In conclusion, as a well-known spice, *C. cassia* barks have been well investigated in the past years. However, whether it could be used for the prevention of DN still needing further investigation. This study resulted in the isolation and identification of three new phenolic glycosides, along with 15 known compounds from the polar extract, of which 11 were isolated for the first time in this species. Compounds **4-8** could significantly inhibit over secretion of fibronectin, collagen IV, and IL-6 against high-glucose-induced mesangial cells at 10 µM, while the other compounds were inactive at 10 µM. Moreover, no cytoxicity was observed at the same concentration by using the MTT assay (Figure 6). These results suggested that *C. cassia* barks contains anti-diabetic nephropathy agents which may be beneficial for DN patients.

## Supplementary Materials

Supplementary materials can be accessed at: http://www.mdpi.com/1420-3049/18/9/10930/s1.
