# Supplementary material for: Identification of Compounds from the Water Soluble Extract of Cinnamomum cassia Barks and Their Inhibitory Effects against High-Glucose-Induced Mesangial Cells
Source: Molecules. 2013 Sep 5;18(9):10930–43. doi: 10.3390/molecules180910930 (PMC6270337; doi:10.3390/molecules180910930)

Supplementary Materials

Contents

S1. ^{1}H-NMR spectrum of compound **1** in DMSO-d_6.

S2. ^{13}C-NMR spectrum of compound **1** in DMSO-d_6.

S3. HSQC spectrum of compound **1** in DMSO-d_6.

S4. HMBC spectrum of compound **1** in DMSO-d_6.

S5. ^{1}H-^{1}H COSY spectrum of compound **1** in DMSO-d_6.

S6. ROESY spectrum of compound **1** in DMSO-d_6.

S7. ^{1}H NMR spectrum of compound **2** in CD_3OD.

S8. ^{13}C-NMR spectrum of compound **2** in CD_3OD.

S9. HSQC spectrum of compound **2** in CD_3OD.

S10. HMBC spectrum of compound **2** in CD_3OD.

S11. ^{1}H-^{1}H COSY spectrum of compound **2** in CD_3OD .

S12. ROESY spectrum of compound **2** in CD_3OD.

S13. ^{1}H-NMR spectrum of compound **3** in CD_3OD.

S14. ^{13}C-NMR spectrum of compound **3** in CD_3OD.

S15. HSQC spectrum of compound **3** in CD_3OD.

S16. HMBC spectrum of compound **3** in CD_3OD.

S17. ^{1}H-^{1}H COSY spectrum of compound **3** in CD_3OD.

Figure S1. ^{1}H-NMR spectrum of compound **1** in DMSO-d_6.

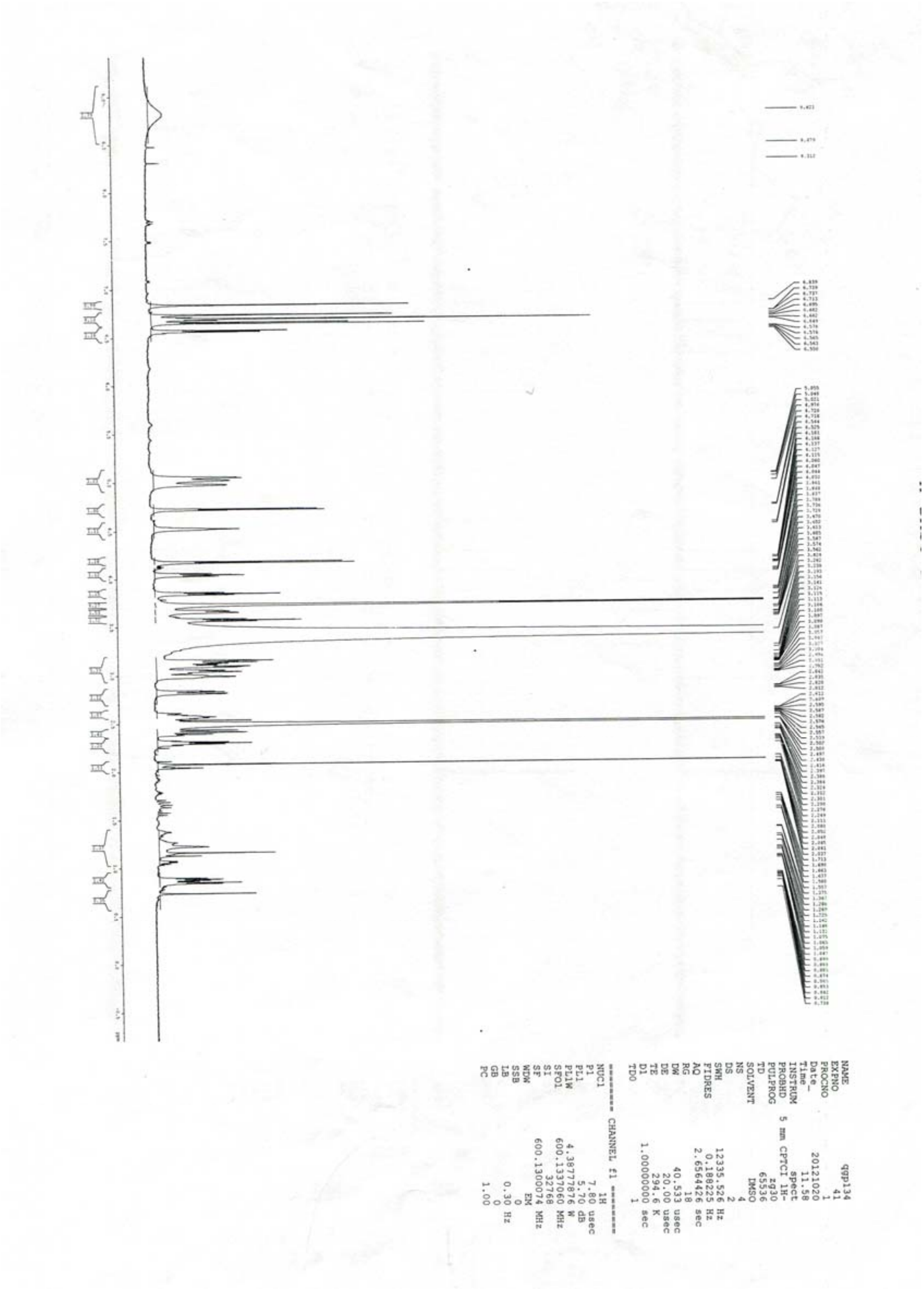

Figure S2. ^{13}C-NMR spectrum of compound **1** in DMSO-d_6.

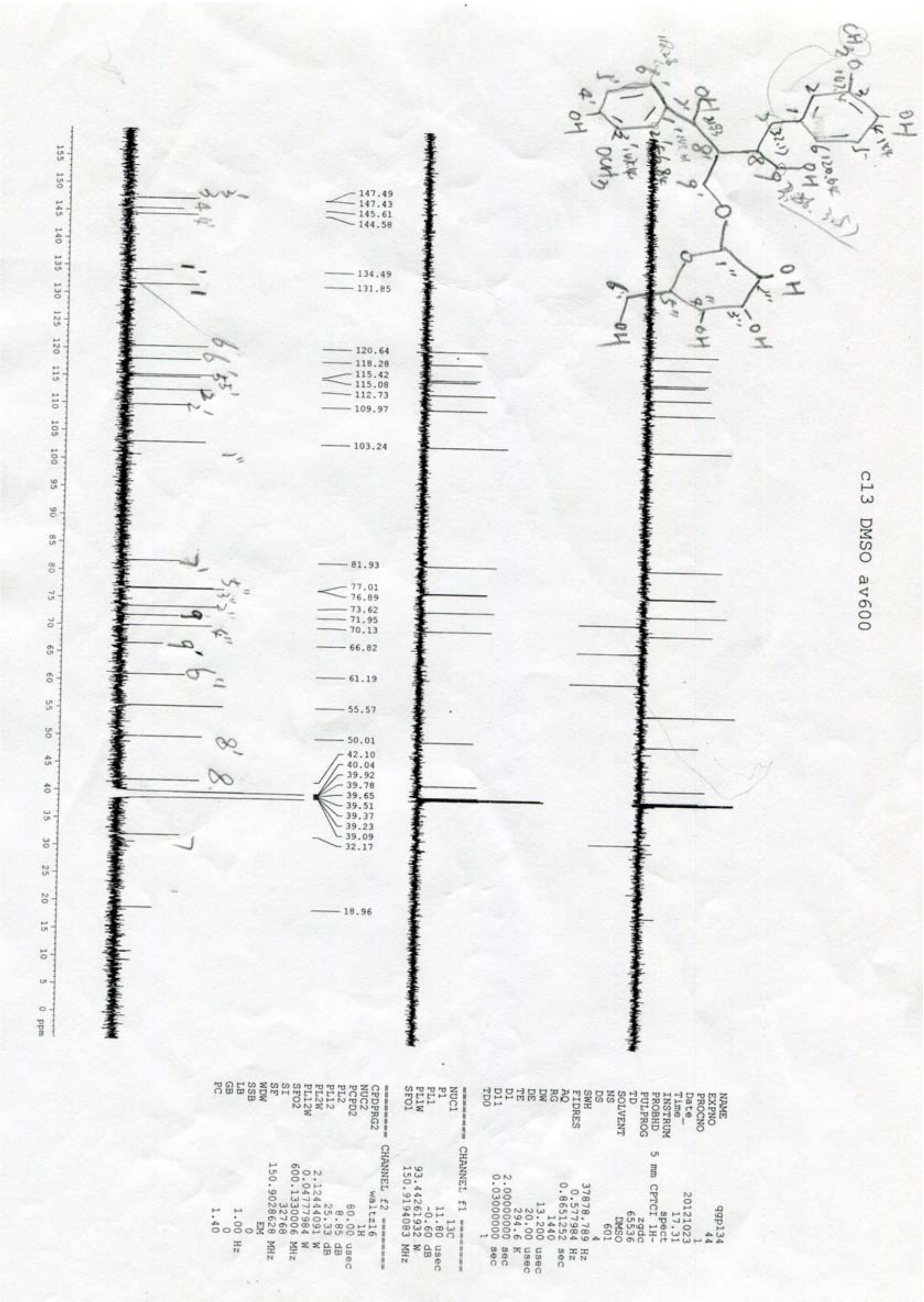

Figure S3 HSQC spectrum of compound **1** in DMSO-d_6

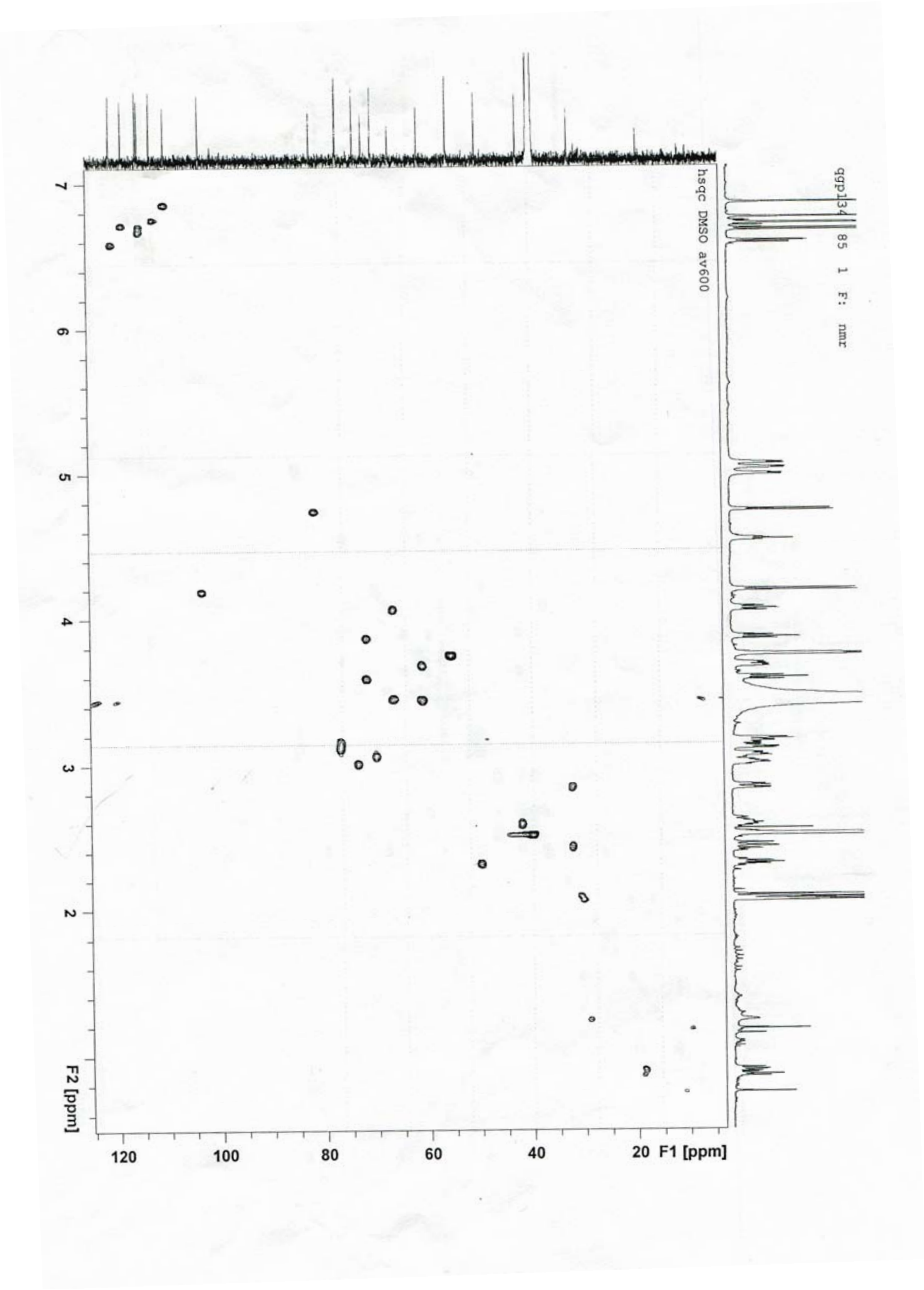

Figure S4. HMBC spectrum of compound **1** in DMSO-d_6.

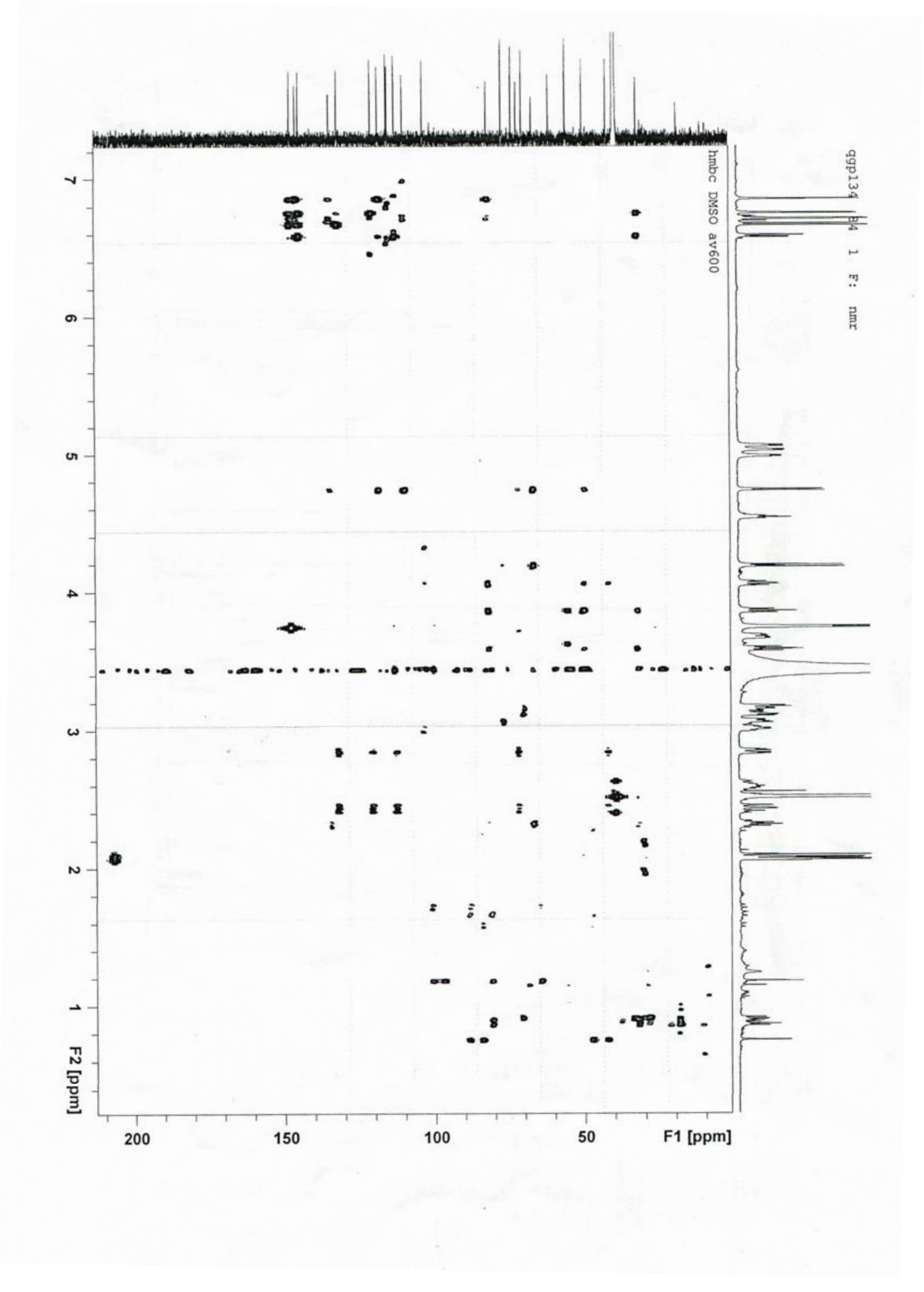

Figure S5. ^1H-^1H COSY spectrum of compound **1** in DMSO-d_6.

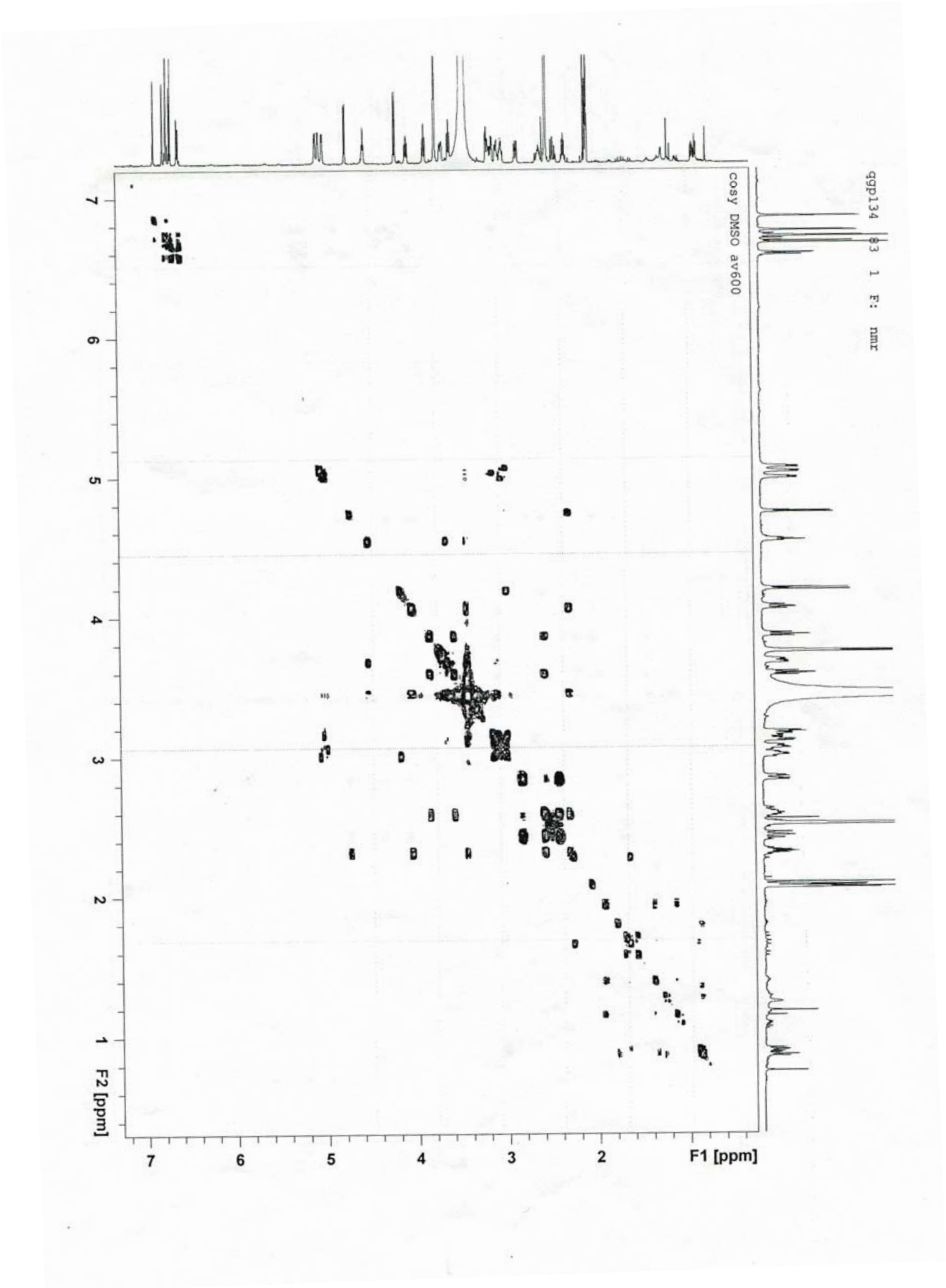

Figure S6. ROESY spectrum of compound **1** in DMSO-d_6.

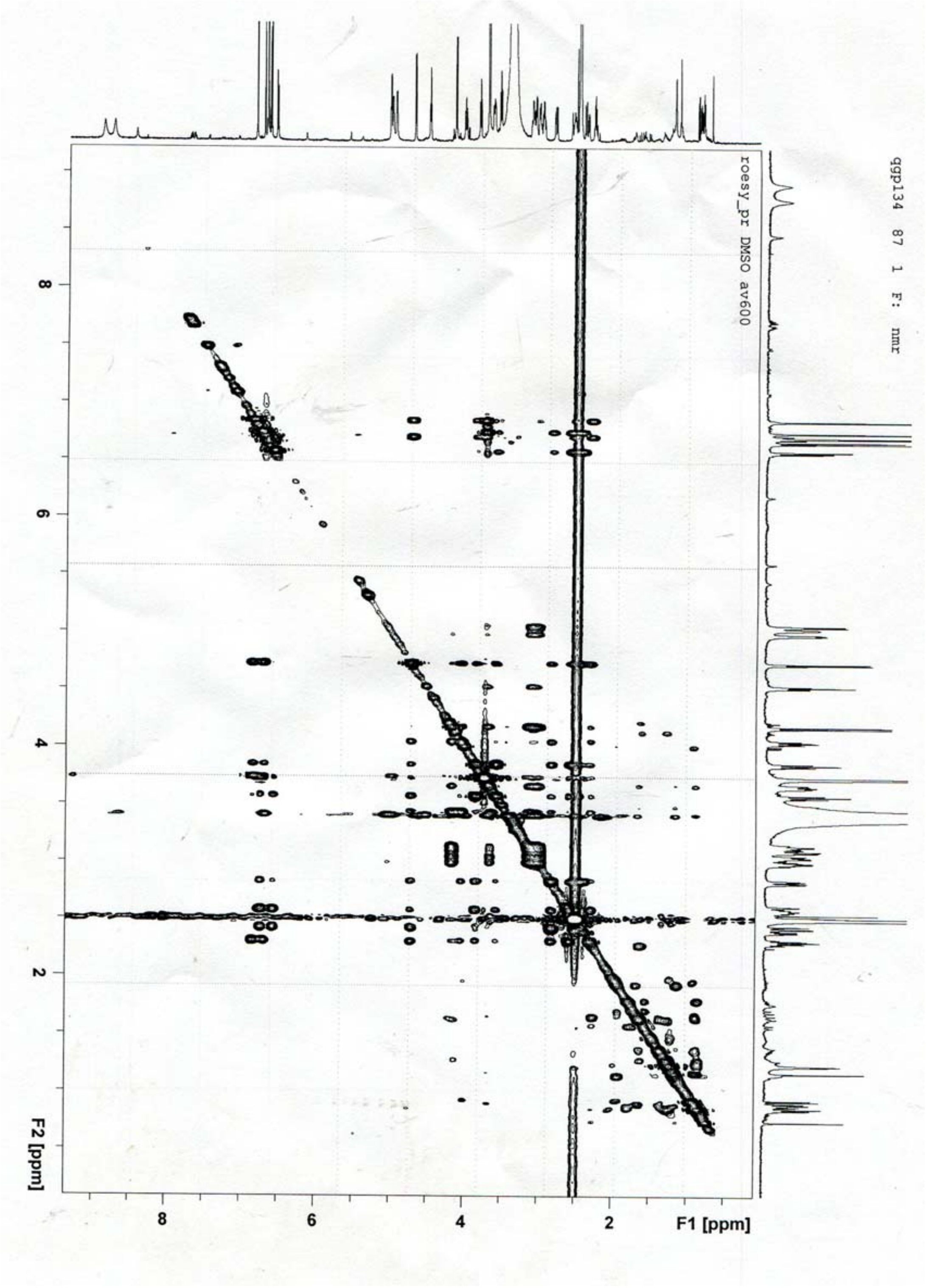

Figure S7. ^{1}H-NMR spectrum of compound **2** in CD_3OD.

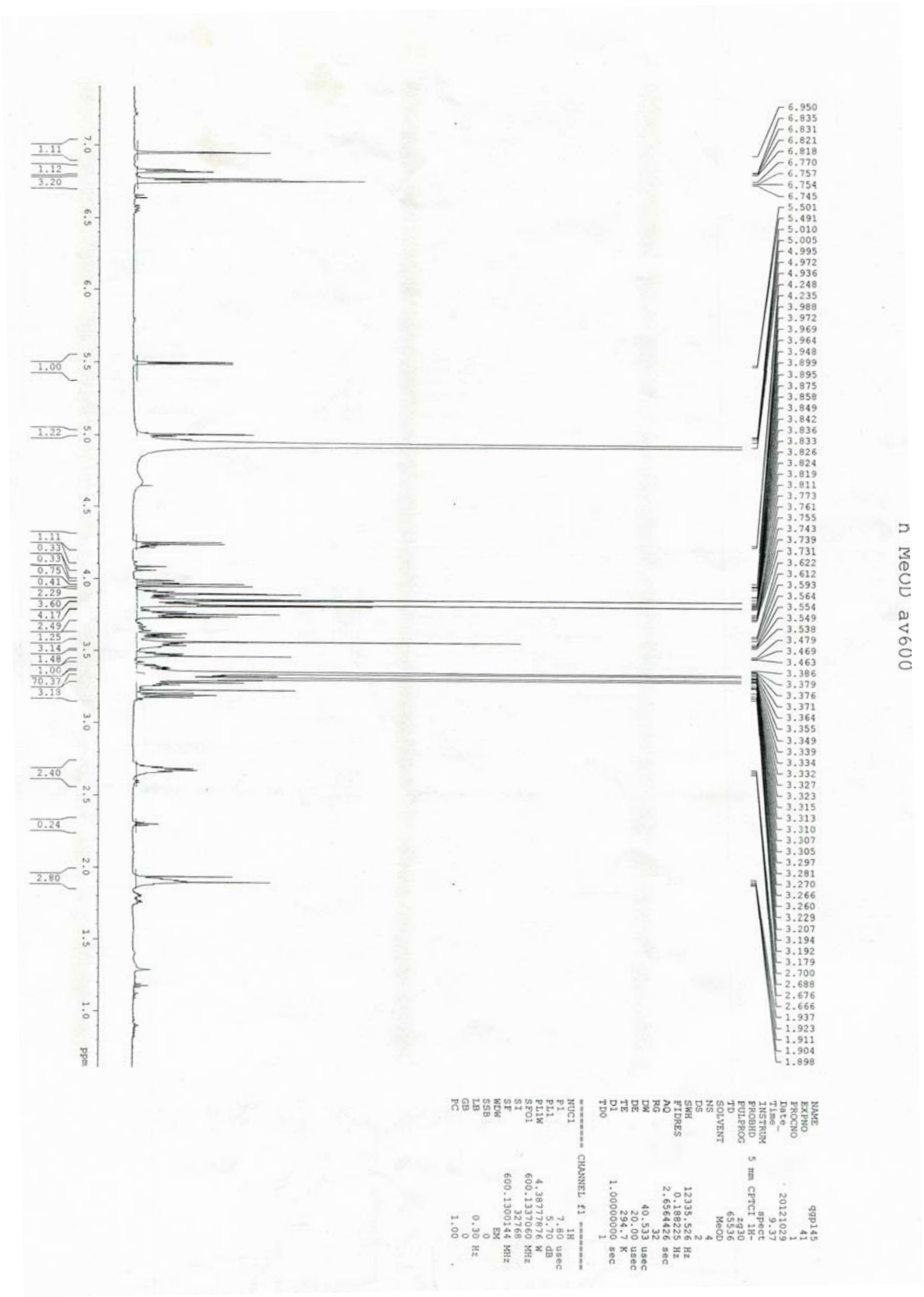

Figure S8. ^{13}C-NMR spectrum of compound **2** in CD_3OD.

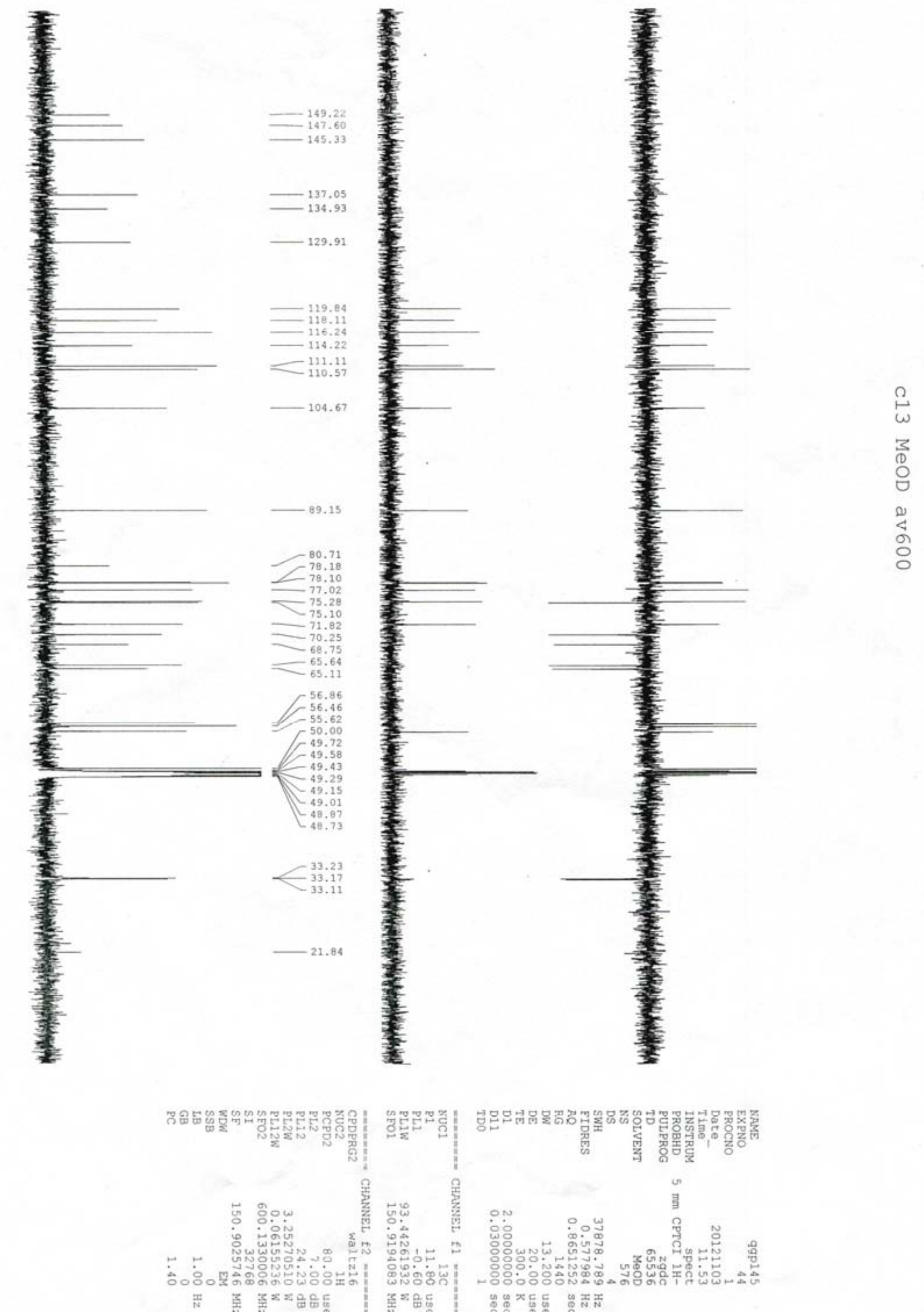

Figure S9. HSQC spectrum of compound **2** in CD_3OD.

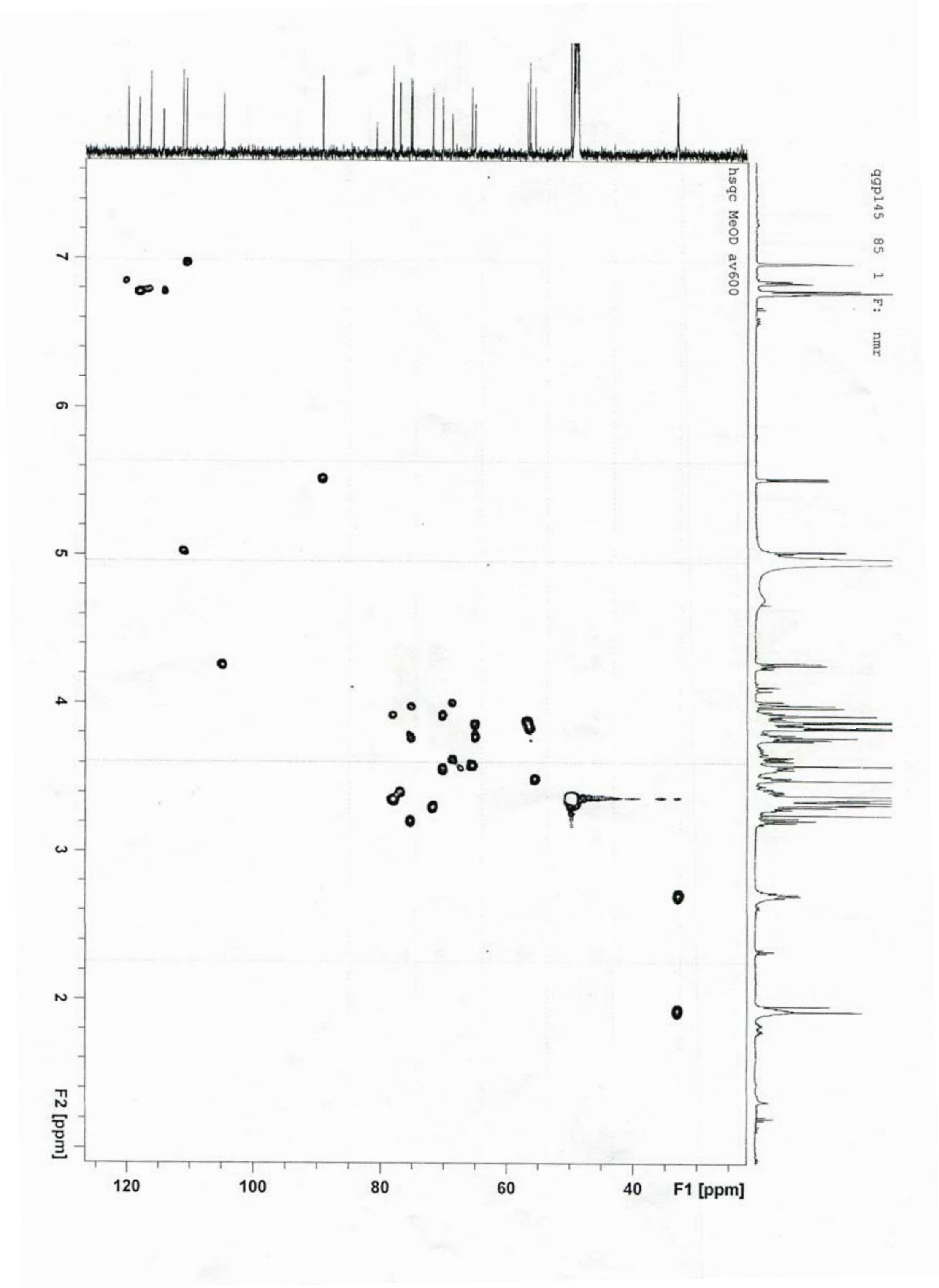

Figure S10. HMBC spectrum of compound **2** in CD_3OD.

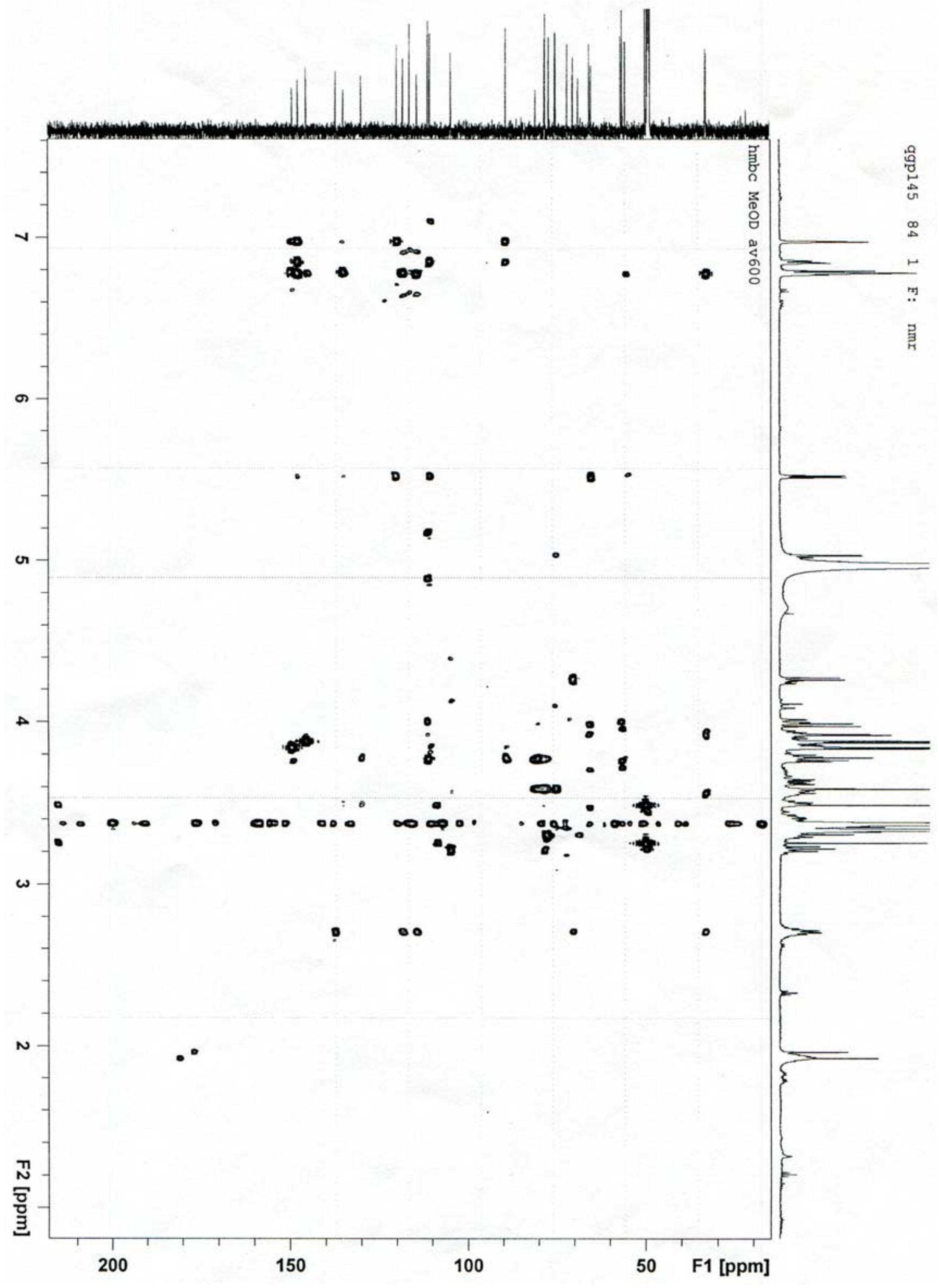

Figure S11. ^{1}H-^{1}H COSY spectrum of compound **2** in CD_3OD.

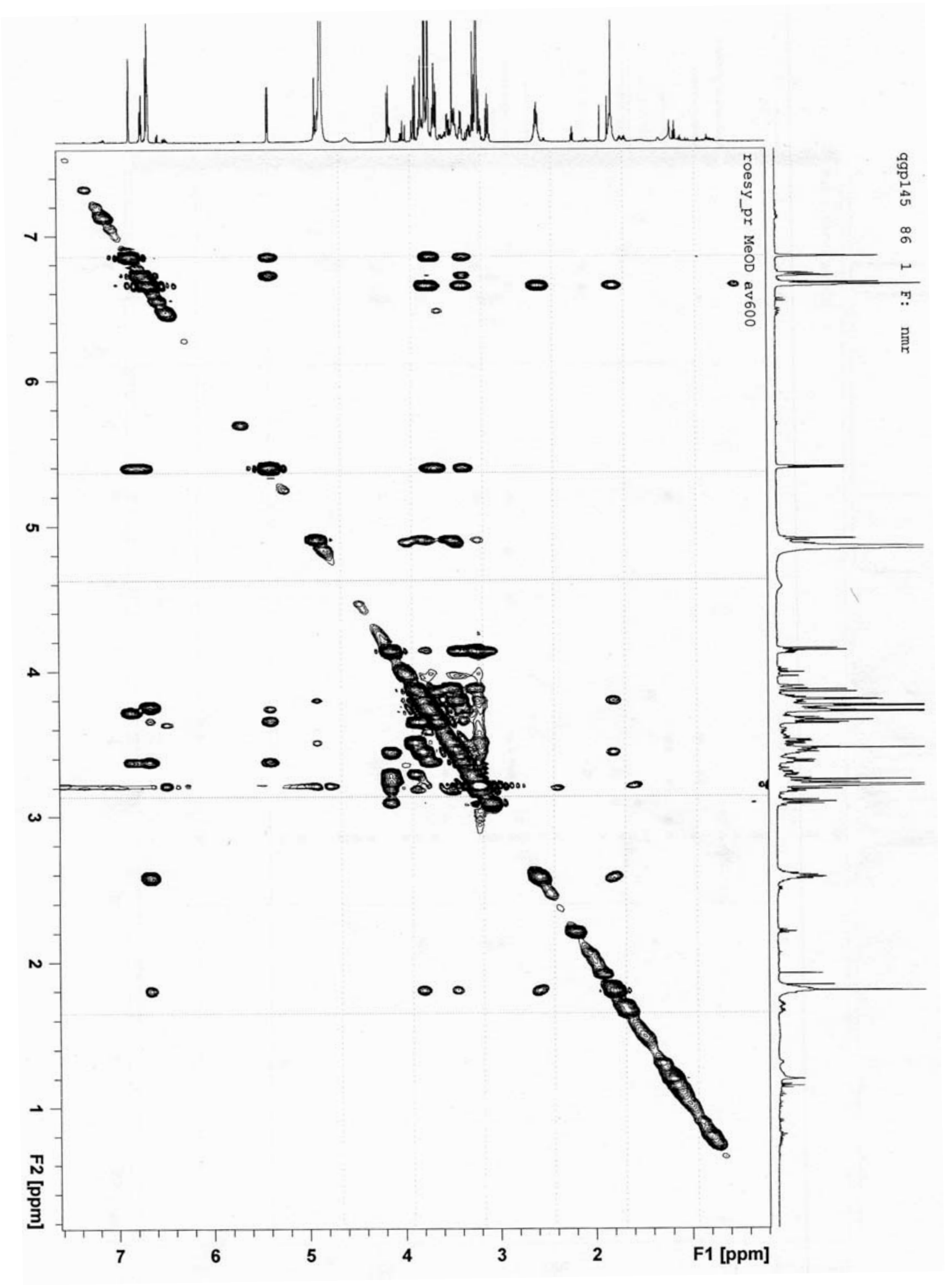

Figure S12. ROESY spectrum of compound **2** in CD_3OD.

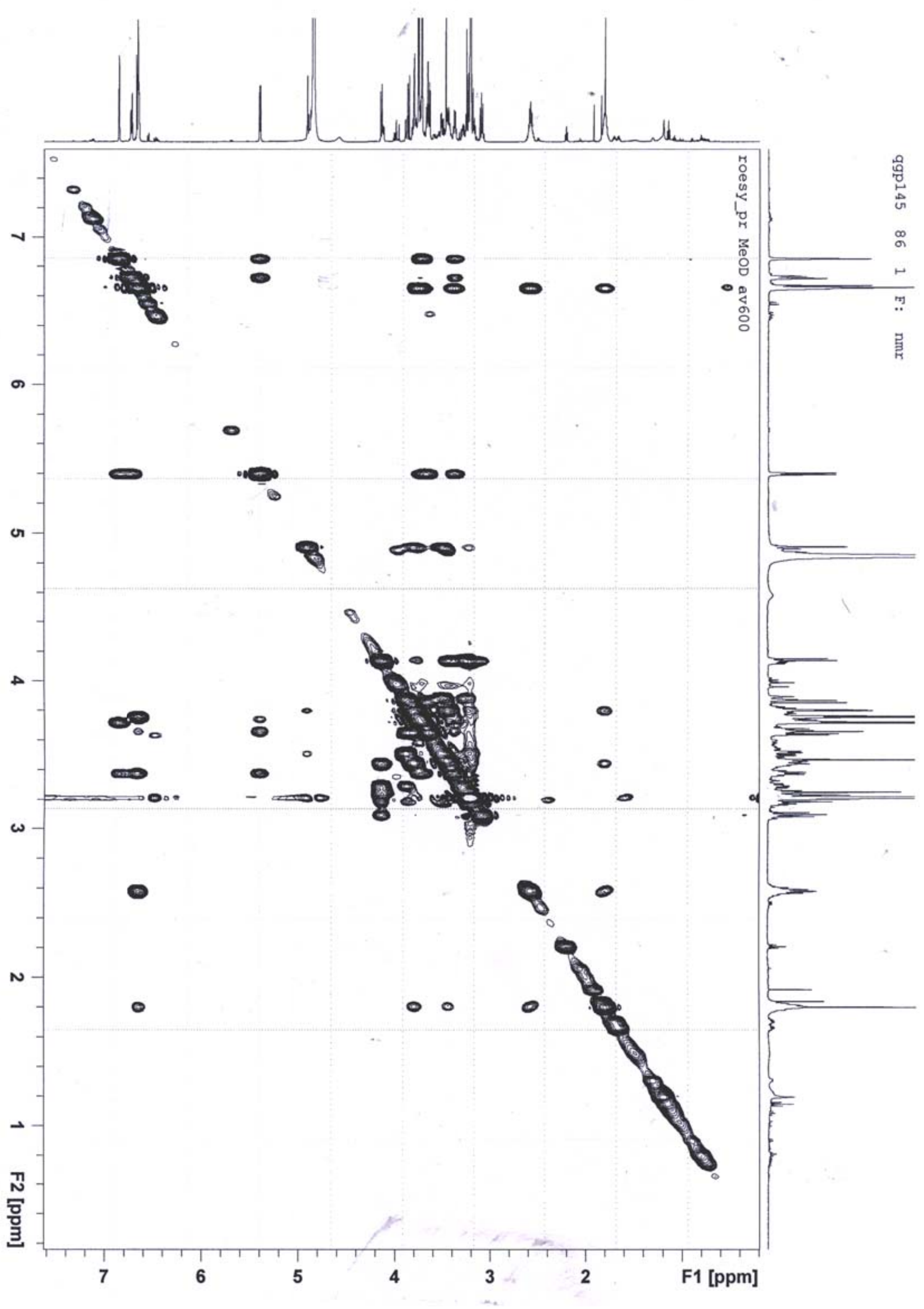

Figure S13. ^{1}H-NMR spectrum of compound **3** in CD_3OD.

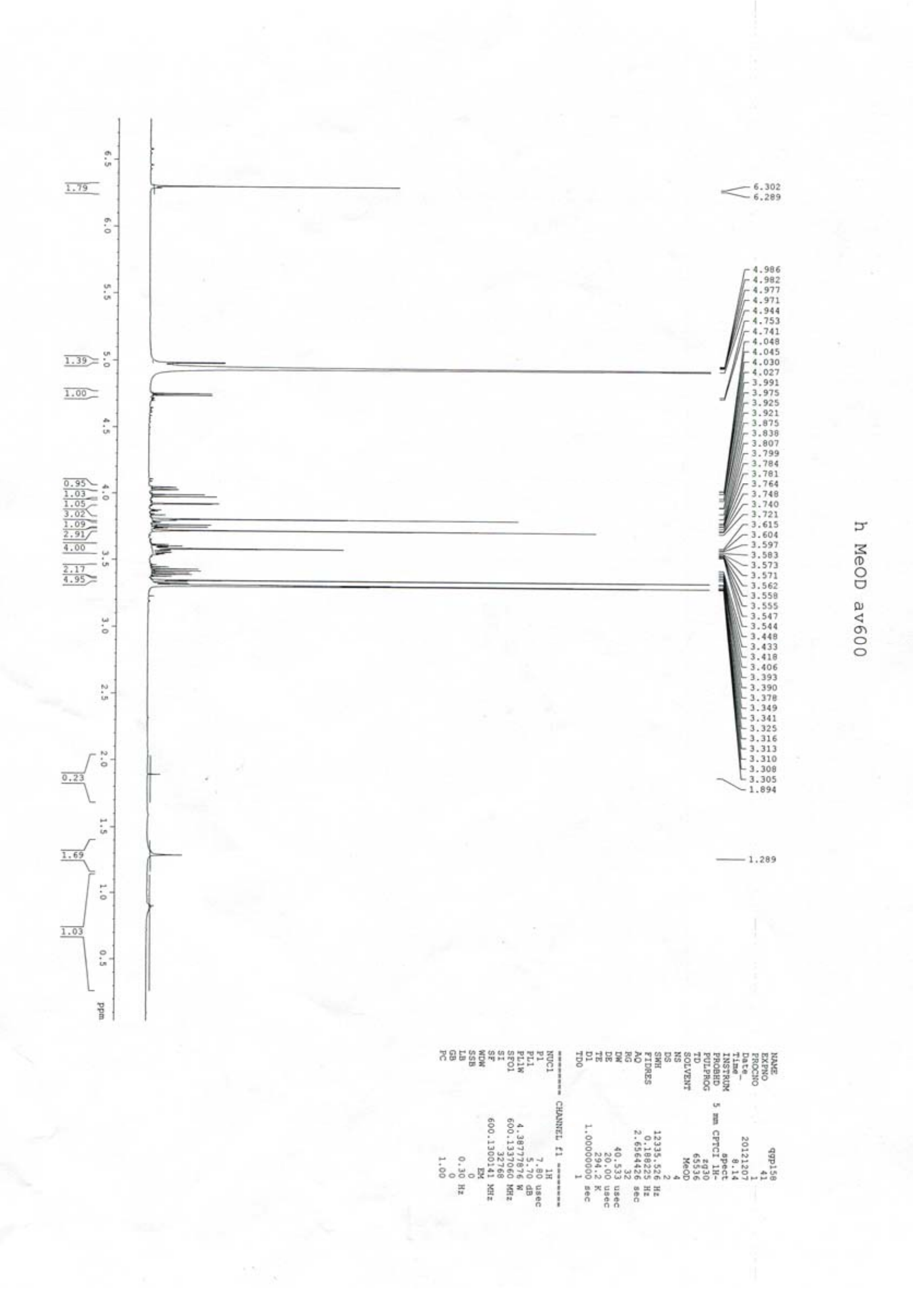

Figure S14. ^{13}C-NMR spectrum of compound **3** in CD_3OD.

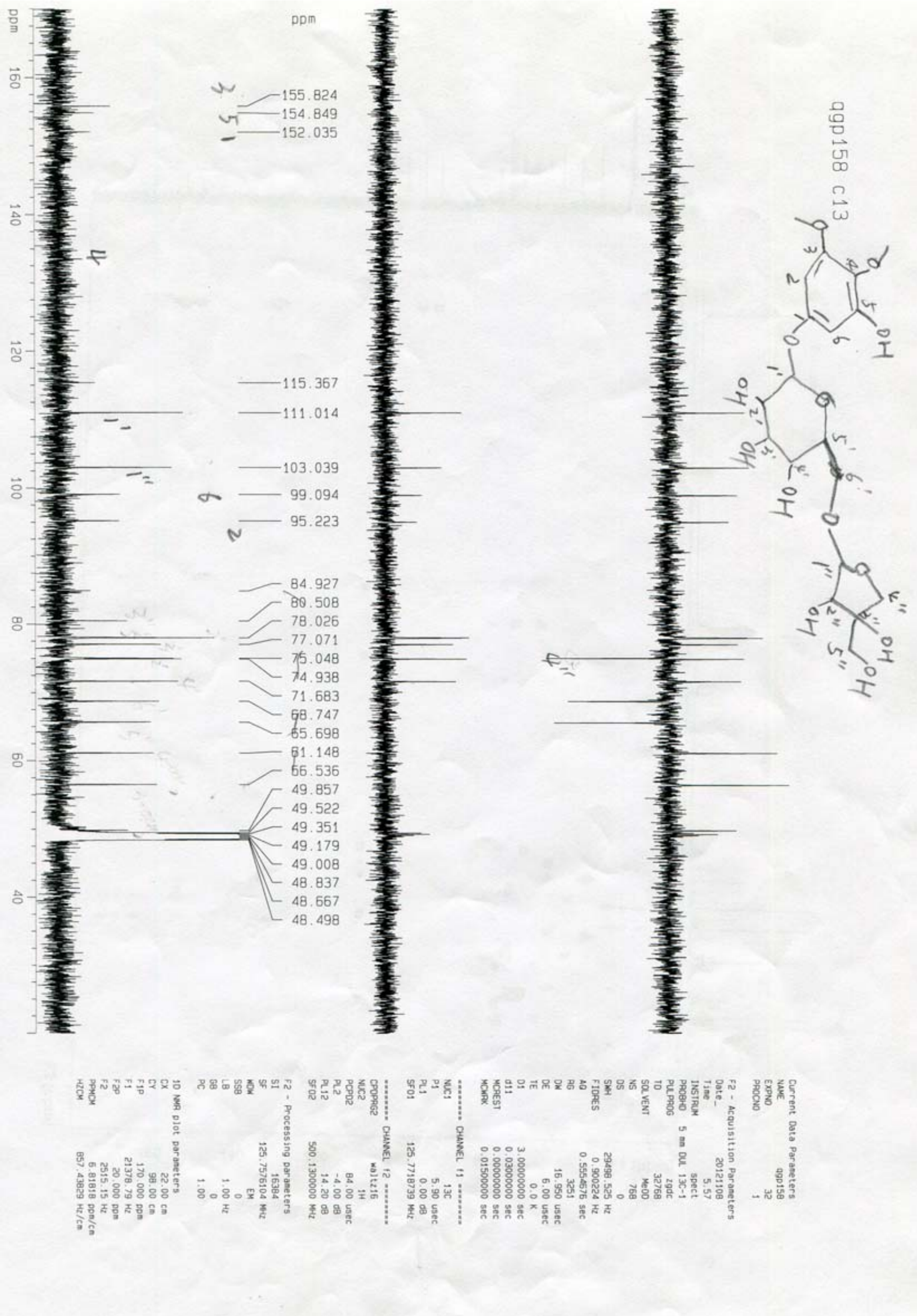

Figure S15. HSQC spectrum of compound **3** in CD_3OD.

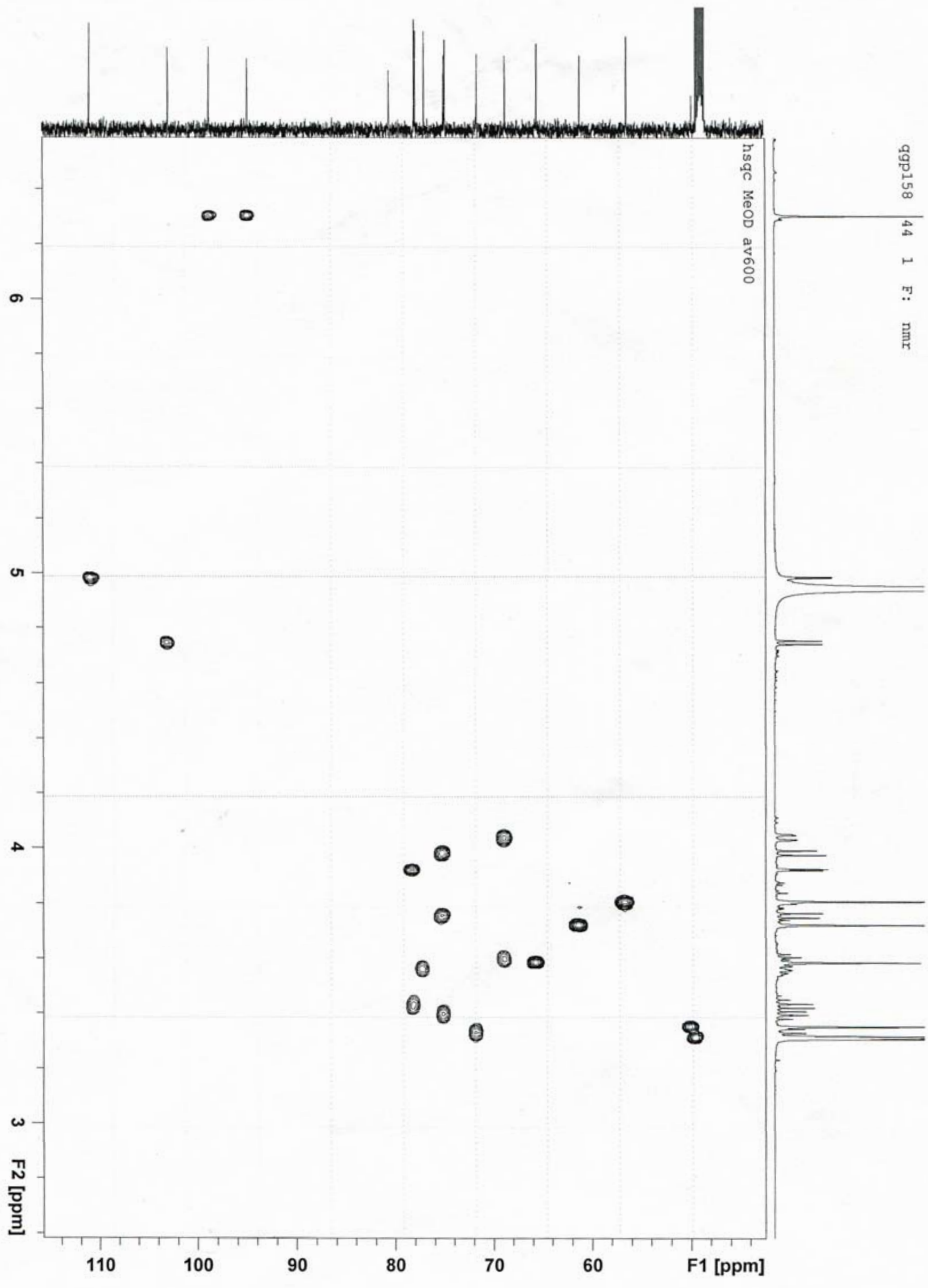

Figure S16. HMBC spectrum of compound **3** in CD_3OD.

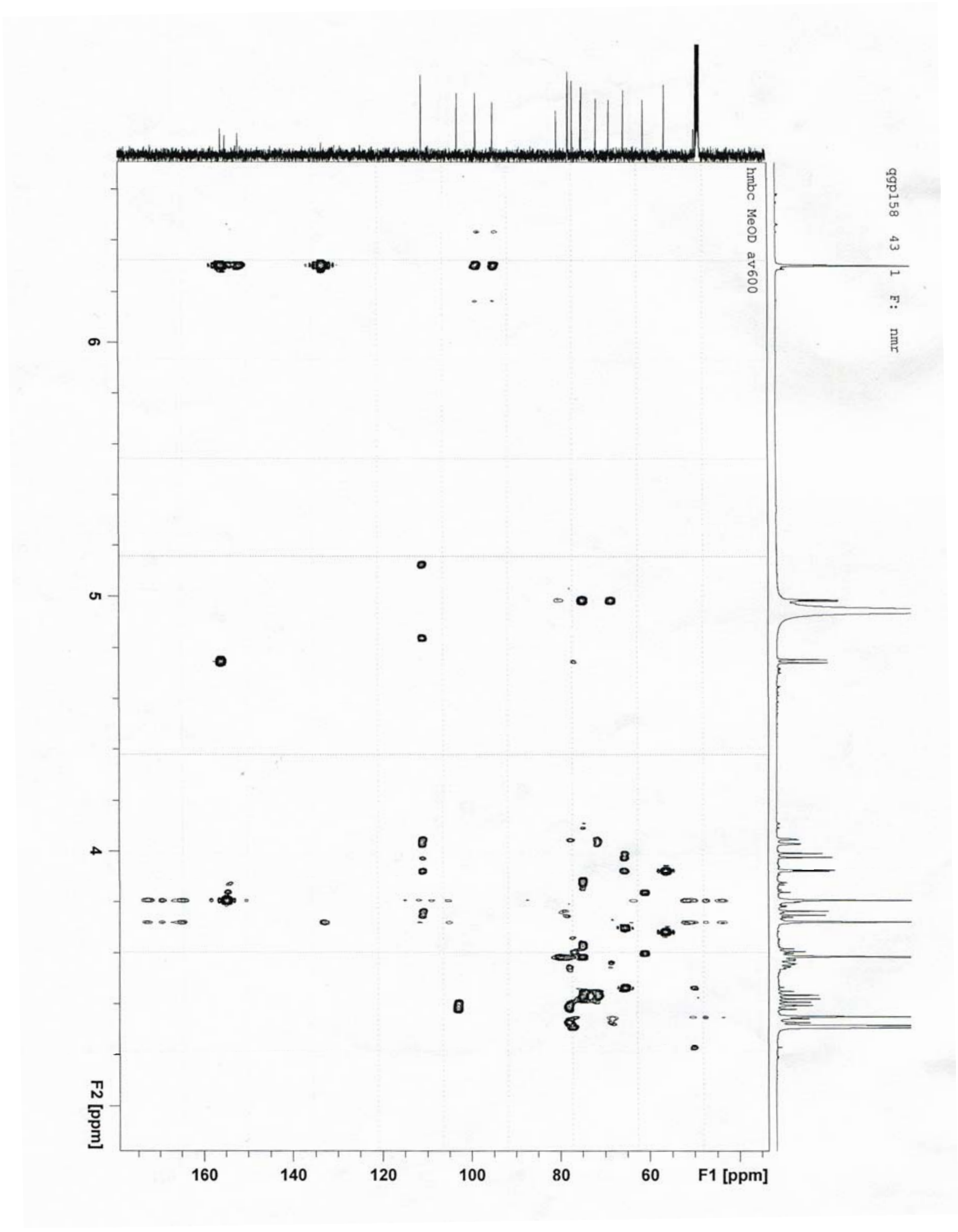

Figure S17. ^1H-^1H COSY spectrum of compound **3** in CD_3OD.

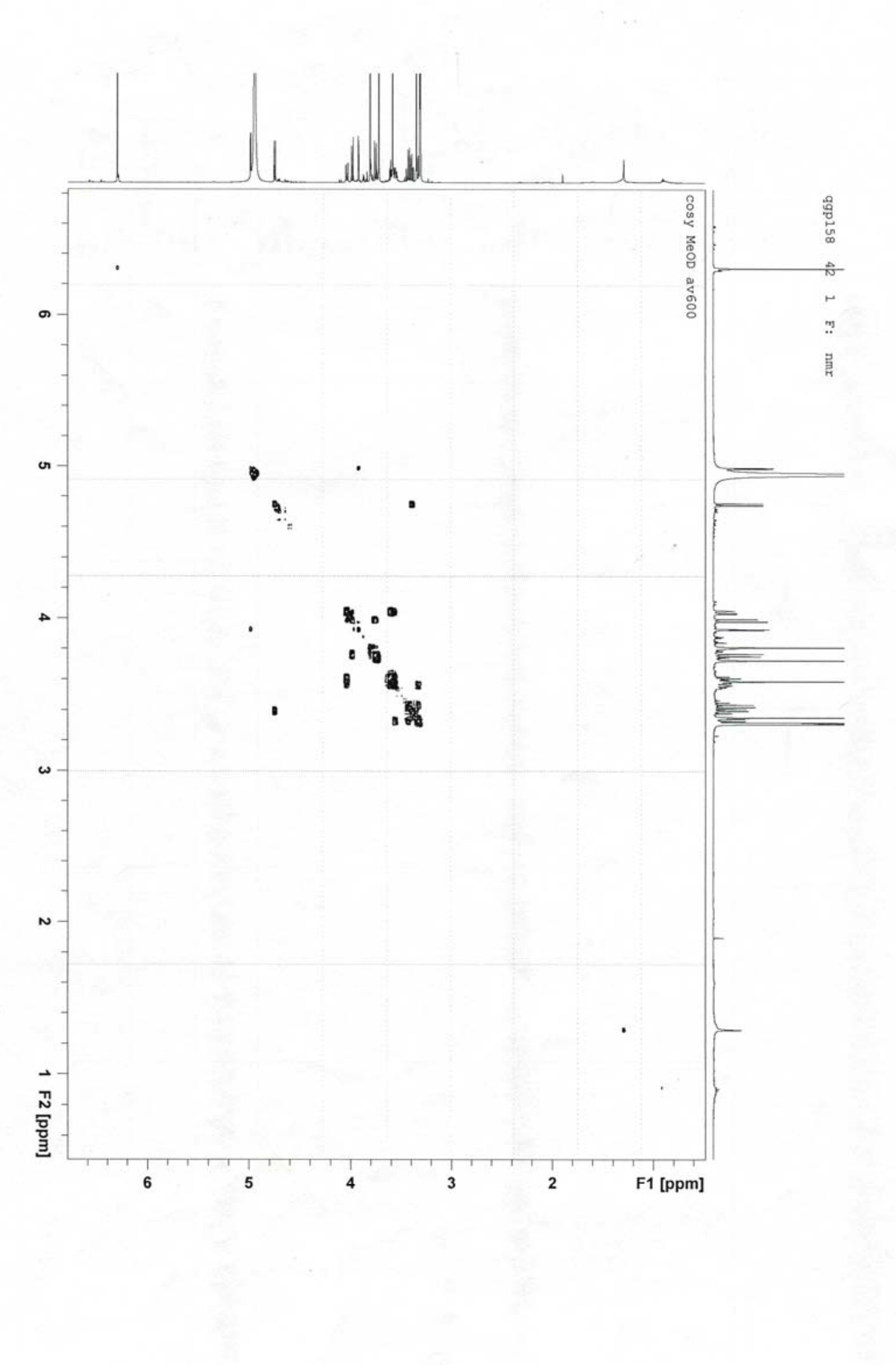

Supplement: Supplementary file 1 [file molecules-18-10930-s001.pdf]
